# Inflammation Based Regulation of Cancer Cachexia

**DOI:** 10.1155/2014/168407

**Published:** 2014-05-04

**Authors:** Jill K. Onesti, Denis C. Guttridge

**Affiliations:** ^1^Division of Surgical Oncology, The Ohio State University Wexner Medical Center, The Ohio State University College of Medicine, 460 W. 12th Avenue, Columbus, OH 43210, USA; ^2^The Arthur G. James Comprehensive Cancer Center, Columbus, OH 43210, USA; ^3^Human Cancer Genetics Program, Department of Molecular Virology, Immunology and Medical Genetics, The Ohio State University, Columbus, OH 43210, USA

## Abstract

Cancer cachexia, consisting of significant skeletal muscle wasting independent of nutritional intake, is a major concern for patients with solid tumors that affects surgical, therapeutic, and quality of life outcomes. This review summarizes the clinical implications, background of inflammatory cytokines, and the origin and sources of procachectic factors including TNF-*α*, IL-6, IL-1, INF-*γ*, and PIF. Molecular mechanisms and pathways are described to elucidate the link between the immune response caused by the presence of the tumor and the final result of skeletal muscle wasting.

## 1. Clinical Significance of Cancer Cachexia

Cachexia associated with cancer leading to skeletal muscle wasting is a major cause of morbidity associated with numerous types of cancer. Varying definitions have been proposed to classify cachexia, but the central components include ongoing loss of muscle mass due to a negative protein balance [1–3]. Greater than 50% of patients with cancer have cachexia at the time of death, and more than 30% of patients die due to cachexia [4]. This has been shown to become increasingly worse as the cancer progresses, eventually reaching a limit with low likelihood of reversal [5]. Emerging evidence shows that skeletal muscle depletion in cancer patients is a powerful predictor of a worse overall prognosis across varying cancer etiologies [6–9].

Muscle atrophy/wasting, often used as a clinical marker of cachexia, has been shown to affect outcomes in patients undergoing surgery. The University of Michigan Analytical Morphomics Group has published their findings on the relationship between lean muscle mass and postoperative mortality in patients undergoing any major elective surgery (an increase in mortality by 45% for each 1000 mm^2^ decrease in lean core muscle area) [9] which they found to be more predictive than chronological age [10]. This same pattern held true for patients with adrenocortical carcinoma [11] and melanoma [12]. The measurements for lean muscle mass were determined by measuring the cross-sectional area and Hounsfield units of the psoas muscle at the level of the fourth lumbar vertebra and excluding fatty infiltration.

Patients with operable cancer are greatly impacted by the presence of cachexia. This may be due to the fact that cachexia indicates a more advanced stage of tumor [13] or simply that the patient is overall frailer. In a study examining 557 patients undergoing pancreas resection for adenocarcinoma, Peng et al. found that muscle wasting was an independent factor associated with an increased risk of death at three years (HR = 1.63; *P* < 0.001) [14]. A similar finding was noted for patients undergoing hepatectomy for hepatocellular carcinoma (HR = 0.92; *P* = 0.004) [15]. Decreased muscle density was associated with an increased rate of complications but not overall outcomes for colon cancer in another study [16].

Not only are overall survival and surgical outcomes affected by cachexia but also quality of life. Several studies have shown that cachexia itself contributes to lower scores more so than tumor location, duration, or stage [17, 18]. Outward effects of cachexia include a decrease in physical activity and the ability to perform activities of daily living, which may play a role in a person's psychological well-being. These factors in turn lead to a lower performance status, negatively impacting the ability and availability of chemotherapeutic agents [18–22].

The theory of the origin of cancer cachexia is rooted in systemic inflammation and not solely reduction of nutritional intake [23], a reason why cachexia is now distinguished from anorexia (see below). Several easily identifiable factors have been studied in an attempt to quantify the degree of inflammation and use that data to predict outcomes or guide treatment. Elevated neutrophil : lymphocyte ratio (NLR) and C-reactive protein have been associated with low skeletal muscle mass [24] and early detection of cancer cachexia [25, 26]. NLR has been shown to predict outcomes in numerous types of solid [27–32]. Another scoring system, the modified Glasgow Prognostic Score, is based on C-reactive protein and albumin levels [33]. This has been shown to predict outcomes in patients with biliary [34], colorectal [35], prostate [36], and other tumors [37].

The effect of trying to reduce cachexia by avoiding bed rest and stimulating muscle exercise has met with limited results. A review by Stene and colleagues [38] summarized results from several randomized controlled trials examining cancer cachexia. They found that physical exercise may lead to reduced fatigue, improved quality of life, and decreased adverse effects, but further studies are needed to identify if there is any survival advantage, particularly in more advanced cancer stages.

## 2. Background of Inflammatory Cytokines

The clinical significance of cancer cachexia has been realized for some time. The imbalance between adequate caloric intake and total body energy expenditure has been the subject of research for several decades. Previous work has focused on the role of cytokines such as tumor necrosis factor-*α* (TNF-*α*), interleukins 1 and 6 (IL-1, IL-6), and interferon gamma (INF-*γ*).

A review article by Tisdale published in 1997 summarized the current literature at that time [39]. Cancer cachexia was noted to be different from simple starvation which strives to conserve muscle mass. In cancer cachexia, however, this conservation mechanism is missing, such that there is equal loss of adipose and muscular tissue. This finding highlights the fact that anorexia alone is not sufficient cause for cachexia, and, in fact, does not always precede it [40], nor is cachexia alleviated by the supplementation of intravenous hyperalimentation [41].

Probably more influential in the development of cachexia is the increase in energy expenditure due to an elevated basal metabolic rate [39]. This is associated with an elevated adrenergic state [42] and appears to be similar across tumor types. Many solid tumors have also been shown to have significantly elevated rates of carbohydrate metabolism [43, 44]. This increase in glucose utilization by the tumor translates into a lower supply for the host tissue. The primary site of lean body mass depletion is the skeletal muscle and this is due to an increased rate of protein turnover without an appropriately significant increase in protein synthesis [45]. The pathway regulating protein breakdown is the adenosine triphosphate- (ATP-) ubiquitin-dependent pathway. This has been shown to be upregulated in cancer cachexia [46] and the ATP-ubiquitin-dependent pathway appears to play a major role in cancer cachexia in weight loss up to 20% [47].

These responses are controlled, at least in part, by a variety of cytokines. TNF-*α* was initially thought to play a direct role in cachexia by inhibiting lipoprotein lipase and enhancing the protein degradation. The direct correlation between TNF-*α* levels and the degree of cachexia has been more difficult to prove, however [39]. Similarly, IL-1 has demonstrated some role in the cachexia pathway, but a direct mechanism for controlling tissue wasting has not been proven [39]. Increasing the levels of IL-6 has been shown to correlate with development of cachexia in certain mouse models [48, 49]. Treatments designed to bind to IL-6 and inhibit its effect have demonstrated improvement in cachexia [50, 51]. These results have also been demonstrated in human patients [52]. Studies have shown INF-*γ* to have similar properties to TNF-*α* in reducing body fat, but without an effect on total body protein [39]. Again, no association with the human clinical syndrome of cancer cachexia has been clearly elucidated.

The ubiquitin pathway is also regulated by a high affinity activin type 2 receptor (ActRIIB) [53]. Zhou et al. found that blockade of this pathway could reverse muscle loss and also led to prolonged survival in mice models of cancer cachexia. Interestingly, this reversal was not accompanied by a reduction in circulating levels of proinflammatory cytokines [53].

In a review by Argiles and Lopez-Soriano, cytokines are separated according to their function as either procachectic factors or anticachectic factors in order to further define their roles [54]. The procachectic factors include those mentioned above, which act by promoting tissue wasting. The anticachectic factors act in opposition by attempting to stabilize this breakdown. These factors include IL-4, IL-10, IL-12, IL-15, INF-*α*, and insulin-like growth factor I (IGF-I). These cytokines have been shown to ameliorate the effects of the procachectic factors to varying degrees, mostly in mouse models [54]. Clearly a balance must exist, and both procachectic and anticachectic factors are targets for clinical therapies.

## 3. Origins of Cachexia Mediators

Once the presence and function of cytokines in the pathogenesis of cachexia has been established, the origin and sources must be identified. Previous theories of the origin of cytokines have included the tumor itself versus the native host tissue [55].

Evidence for the release of cytokines from native host tissue is found in the presence of a persistent inflammatory response, mediated by T helper 1 (Th1) cells [55]. The presence of the tumor itself causes the body to produce an acute phase response [56]. A review by de Visser and Coussens described how the body's innate immune system involves an increase in the local concentration of mast cells and macrophages leading to angiogenesis and tumor growth [57]. Mouse models of epithelial carcinogenesis have demonstrated that the absence of mast cells or the inability to recruit additional immune cells prohibits malignant transformation [58]. Macrophages appear to be the source of some of the principal mediators of cachexia, such as TNF-*α* or IL-1 [59]. Intriguingly, chronic inflammation may be associated with compromised immune function, such as an impaired T-cell response, via various inflammatory proteins, including sIL-2R, VEGF, and IL-17 [60], thus creating an environment even more permissive to tumor survival.

Certain myeloid immune suppressor cells have been found to promote tumor angiogenesis by the production of matrix metalloproteinase 9 (MMP-9) [61]. These factors even suggest that the presence of host immune cells is required for promoting neoplastic events [57]. Tumor infiltrating inflammatory cells also regulate angiogenesis as well as producing extracellular proteases that serve to remodel the extracellular environment allowing tumor potentiation and possibly even metastases [57, 62]. The authors make note that expression of MMP-9 primarily derives from host immune cells such as neutrophils, macrophages, and mast cells, as opposed to tumor cells [62].

One study found that a population of myeloid-derived suppressor cells grows dramatically within tumors, producing inappropriate quantities of inflammatory cytokines [63]. This increase was noted to be associated with cachexia. These cells and others of the innate immune system respond to tumors by producing TNF-*α*, IL-1*β*, IL-6, and INF-*γ* in an effort to stimulate the host's immune response and overcome any offending pathogens. As the cancer persists, however, the ongoing high inflammatory state begins to have ill effects towards the host, as well.

The specific role of IL-6 in cancer associated cachexia and skeletal muscle wasting has been identified [64]. In a study by White and colleagues, *Apc* ^*Min*⁡/+^ and wild type mice on a C57Bl/6 background were used to examine the effect of treatment with an IL-6 receptor antibody after the onset of cachexia as well as the effects of exercise [65]. They found that mitochondrial biogenesis was disrupted early in the development of cachexia, which could be rescued by administration of an IL-6 receptor antibody as well as exercise. Which factors downstream of IL-6 mediate effects on cachexia are still being elucidated but likely involve the transcription factor STAT3, which we describe in more detail below.

Tumor specific factors include proteolysis inducing factor (PIF) and lipid mobilizing factor (LMF), which serve to direct breakdown proteins and fat [55]. Increased concentrations of PIF have been identified in murine models consistent, and almost exclusively, with cancer cachexia [66] likely through the ATP-ubiquitin-dependent pathway [67]. In a study examining a human homologue of PIF, however, although elevated levels were noted in the presence of tumor, this alone was not enough to induce cachexia [68]. Another study found that PIF was expressed in patients with gastrointestinal tumors and that this expression correlated with weight loss [69].

The specific role of the tumor versus the host response is not always clearly delineated. Procachexia cytokines might be produced by the tumor as well as the host, whereas PIF appears to be produced exclusively by tumors [70]. In addition, PIF and TNF-*α* appear to induce muscle cachexia through a similar pathway, by activating the nuclear factor kappa B (NF-*κ*B) transcription factor [71, 72]. Activation of this factor causes translocation to the nucleus where it binds to specific promoter regions, regulating the expression of proinflammatory cytokines [55] as well as the ubiquitin-proteasome pathway. Another pathway responsive to inflammation that was recently implemented in regulation of the ubiquitin-proteasome system is the CCAAT/enhancer binding protein beta (C/EBP*β*) transcription factor whose activation depends on p38 MAP kinase.

Although PIF appears to clearly contribute to skeletal muscle loss in cancer cachexia, no other purely tumoral factor appears to have the same potential [70]. Therefore, the majority of mediators are due to the host's systemic response.

Another pathway that may contribute to cancer cachexia is autophagic degradation. The host's natural autophagic-lysosomal proteolysis may be altered in various pathologic states. In a study by Mizushima et al. autophagy was enhanced in skeletal muscle during the first 24 hours of starvation and sustained [73]. A direct link has also recently been described in cancer cachexia models, which showed that increased autophagic-lysosomal degradation is induced in cancer associated muscle atrophy and likely involves separate pathways from those involved in noncancer muscle wasting [74]. The FoxO transcription factors have been shown to function as strong transcriptional drivers of autophagic genes in response to cachectic factors [75].

## 4. Genetic Response to Cytokine Stimulation: STAT3 and Pax7

As described above, cytokines are important not only to establish tumor-host interaction and deregulate inflammatory response to tumor burden but also as mediators of muscle wasting by directly targeting muscle tissue. To this regard, cachexia appears to be a genetically regulated response, dependent on a specific subset of genes, which control a highly regulated process of muscle protein degradation [76]. Bonetto et al. described the process by which STAT3 is activated leading to an upregulation of the acute phase response [77]. IL-6 binds to the IL-6 reception *α*-chain, which causes dimerization and activation of associated Janus kinases. Two pathways are then activated, the STAT3 and the mitogen-activated protein kinase (MAPK/ERK) cascade. STAT3 then causes further dimerization and nuclear translocation and ultimately modulation of gene expression of the acute phase response. In their study, Bonetto et al. implanted colon-26 adenocarcinoma cells into Balb/c or CD2F1 mice. Mice were sacrificed after 19 and 24 days (10 and 15% weight loss, resp.) reflecting moderate and severe cachexia. Significant STAT3 activity was noted in gastrocnemius and quadriceps muscles. Mice were then injected with a recombinant adenovirus that constitutively expressed STAT3 and found significant elevation of fibrinogen levels, indicating that IL-6 activation of STAT3 is a potent stimulator of the acute phase response that leads to significant cachexia. It is worth noting that the authors found a low level of suppressor of cytokine signaling-3 (SOCS3) in this tumor model, which normally serves to inhibit STAT3 and self-regulate the duration of activation. This could explain how cachexia continues to persist despite clearly deleterious effects on the host.

STAT3 activation is not isolated to the IL-6 pathway, however. PIF has also been shown to activate STAT3 in hepatic cells, which also increases the production of proinflammatory cytokines leading to cachexia [78]. PIF has no other known function other than muscle degradation, but the authors theorize that its function could be critical during embryogenesis. Expression peaks during skeletal muscle and liver development in the developing fetus.

We and others have reported the observation of a massive upregulation of the muscle stem cell specification gene Pax7 in experimental models of cancer cachexia [79, 80]. Penna et al. inoculated Balb-c mice with colon-26 undifferentiated carcinoma. One group of mice was then injected with the MEK inhibitor PD98059. The mice were allowed free access to food and were sacrificed after 13 days. Significant muscle and body weight loss were observed, as was marked the phosphorylation of ERK, a mitogen activated protein kinase. Evidence for impaired myogenesis was noted in the tumor-bearing mice as evidenced by increased levels of Pax7. The degree of muscle wasting and Pax7 concentration were ameliorated by the injection of the MEK inhibitor PD98059, via inhibition of ERK. These findings supported the idea that satellite cells accumulate in muscle due to overproduction or impaired differentiation, leading to cachexia [79]. Similarly, elevated levels of Pax7 were found in skeletal muscle samples from patients with pancreatic cancer demonstrating cachexia [80]. This overexpression was shown to cause significant muscle atrophy due a block in the differentiation of muscle progenitor cells responding to injury signals emanating from the tumor. We found that the decreased levels of Pax7 could reverse the effects and allowed progenitor cells to differentiate and myofibers to be repaired [80]. Yet to be identified factors present in the serum of tumor-bearing mice are responsible for Pax7 upregulation and block of myogenic potential in muscle stem cells, a capacity not fully recapitulated by administration of specific, albeit important, recombinant cytokines, such as TNF-alpha [80]. This study not only pointed out for the first time the involvement of muscle stem cells in muscle wasting that does not merely consist of muscle fiber atrophy but also demonstrated that circulating factors have multiple targets in muscle and further extend their role in muscle homeostasis. Intriguingly, NF-*κ*B was known for its role in response to inflammatory cytokines in many cell types including muscle [81, 82] and was previously demonstrated to be sufficient to trigger muscle atrophy [83, 84].

## 5. Clinical Trials

Several trials have been performed to identify the physiologic and clinical results of anticachexia treatment modalities in patients with advanced cancer. MacCiò et al. treated patients who had gynecological cancers with megestrol acetate plus l-carnitine, a COX-2 inhibitor (celecoxib), and antioxidants versus just megestrol acetate alone [85]. The combination treatment resulted in improvements in lean body mass, resting energy expenditure, fatigue, and quality of life. Proinflammatory cytokines and oxidative stress markers including IL-6, TNF-*α*, CRP, and reactive oxygen species (ROS) were decreased in the combination arm but were unchanged in the megestrol acetate alone arm.

A phase I/II study compared etanercept (an TNF-*α* blocker) with gemcitabine versus gemcitabine alone for treatment of patients with advanced pancreatic cancer [86]. Some clinical benefit was identified and was associated with IL-10 levels but did not show significant improvement in 6-month progression free survival compared to gemcitabine alone.

Similarly, a phase II trial compared the efficacy and safety of celecoxib on cancer cachexia [87]. All patients had advanced cancer of varying tumor sites. TNF-*α* levels were shown to decrease in the majority, and patients had a corresponding increase in lean body mass. However, changes in IL-6 levels were not significantly different after treatment.

## 6. Conclusions

Cancer cachexia is a very prevalent and debilitating aspect of solid tumors. In addition to predicting an overall worse prognosis, cachexia significantly decreases a patient's quality of life. Surgical outcomes are worsened, chemotherapeutics agents are limited, and daily activities are hindered.

The pathogenesis of cancer cachexia is highly dependent on the patient's immune response. Inflammatory cytokines, procachectic factors, induce muscle degradation even in the face of adequate nutrition. These cytokines are produced by the host in response to the tumor, as well as from tumor factors themselves. IL-6, TNF-*α*, and PIF are major contributors to the syndrome of muscle wasting.

The common pathway for muscle degradation involves the ubiquitin-proteasome pathway. Upstream activation is performed primarily through the NF-*κ*B and STAT3 pathways, making them targets for potential interventions.

More research is essential to further elucidate and halt the dangerous progression of skeletal muscle breakdown in the face of solid tumors.
